# Stability and functionality of bovine lactoferrin powder after 9 years of storage

**DOI:** 10.1016/j.crfs.2025.101036

**Published:** 2025-03-18

**Authors:** Yin Hu, Shubo Luo, Yuhong Jiang, Jie Lin, Baoguo Xu, Zhi-Hong Zhang, Benu Adhikari, Tiantian Xu, Bo Wang

**Affiliations:** a School of Food and Biological Engineering, Jiangsu University, 212013, Zhenjiang, Jiangsu, China; b Heilongjiang Feihe Dairy Co., Ltd, 164800, Harbin, Heilongjiang, China; c Nanjing Bestzyme Bio-Engineering Co., Ltd, 211100, Nanjing, Jiangsu, China; d School of Science, RMIT University, VIC, 3083, Melbourne, Victoria, Australia; e Laboratory Animal Research Center, Jiangsu University, 212013, Zhenjiang, Jiangsu, China

**Keywords:** Lactoferrin, Long-term storage, Antibacterial activity, Antioxidant activity

## Abstract

Bovine lactoferrin (bLF) is a multifunctional protein widely used in food industries. Most bLF products are delivered in a powder form; however, their stability remains unclear. Herein, freeze-dried bLF powders were stored at 4 °C and 40 % relative humidity (RH) for 9 years since 2016. After the long-term storage, their functional properties, including antibacterial ability, antioxidant ability and iron-binding ability, were determined and compared with those of eight commercial LF powders. The bLF powder stored for 9 years demonstrated comparable physicochemical and functional properties with those of commercial LF powders (e.g. >93 % water solubility, >100 mg/100 g iron-binding ability, and >1.7 logCFU/mL bacterial growth reduction against *Salmonella enteritidis*). The haemolysis test indicated that the bLF stored for 9 years exhibited good biocompatibility at a concentration of <5 mg/mL. Therefore, bLF powders can be stored for extremely long periods (>9 years) with minimal side effects. These findings can expand the utilisation of lactoferrin to many specific cases such as voyage and aerospace foods.

## Highlights

• Bovine lactoferrin (bLF) powder was stored at 4 °C for nine years.
• Antioxidant and antibacterial activity of the long-term stored bLF were determined.
• The long-term-stored bLF has good biocompatibility at a concentration of <5 mg/mL.
• The nine-year-stored bLF powder was comparable with commercial bLF powders.

## Graphical abstract

**Figure:**
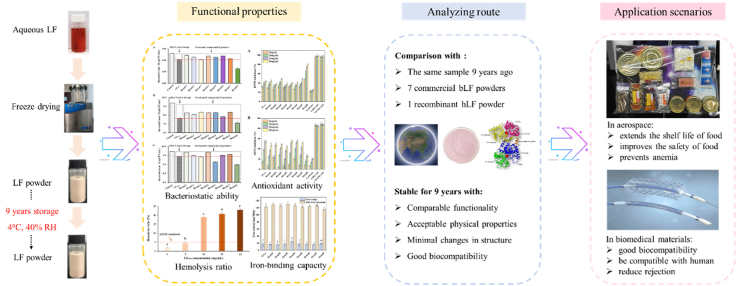

## 1 Introduction

Only ∼40 % of newborns worldwide are fed breast milk during the first 6 months after birth, and <25 % during the first year after birth (Brasfield et al., 2021; Penugonda et al., 2022). Such nutritional deficiency during early life might cause irreversible damage to children's wellness (Lockyer et al., 2021). Therefore, infant formulas are developed and provided to babies to meet their nutritional demand. The ultimate objective of infant formula development is to reach 100 % similarity with breast milk.

Lactoferrin (LF), which is the second most abundant protein in human colostrum, has been attracting considerable attention from dairy industries owing to its multiple functions in infants, such as anti-inflammatory, antibacterial and immunoprotective effects (Sawale et al., 2025). LF is predominantly found in high quantities in human and bovine lactations. In particular, human colostrum exhibits an average LF content that can increase up to 6 g/L, with the concentration gradually decreasing and being maintained at 1–2 g/L. The LF concentrations in bovine colostrum and normal milk are ∼0.8 and 0.03–0.49 g/L, respectively, which are considerably lower than those in the human colostrum and normal milk. With advancements in research, scientists have found that LF present in breast milk and cow milk exhibit similar biological activities and that the amino acid sequence homology between the two reaches 69 % (B. Wang et al., 2019). Therefore, dairy industries are including bovine LF (bLF) into infant formula and other types of nutrient supplemental products.

LF is usually preserved in powder form using spray drying and freeze-drying techniques. In the food industry, spray drying technology is more favored for the production of protein powders, as it can convert liquid feedstock into powder particles, offering scalability and cost-effectiveness compared to freeze drying (Deshwal et al., 2020). Despite the higher cost-effectiveness of spray drying, this technology has not been widely used for the powderization of LF due to insufficient research on the denaturation and other physicochemical properties that may occur during the spray drying process. During the extraction and drying process of proteins, the processing parameters employed can lead to certain variations in the protein structure, which undoubtedly affect the functionality and nutritional value of the proteins. Freeze drying, or lyophilization, can preserve bioactive compounds with minimal damage (Laina et al., 2024). Changes in the structural characteristics and functional properties of LF after freeze- and spray drying processing have been revealed in our previous reported study (B. Wang et al., 2017a). However, changes in the stability and functionality of the freeze- and spray-dried LF powders during storage have not been fully understood. Current literature reports on the stability of LF powders during short-term storage (4 weeks) (Mitsudome, 2024). Data have shown that LF clustering occurs because of localised structural variations and shifts in β-strand interactions, implying that minor structural modifications, as opposed to substantial comprehensive alterations, facilitate the aggregation process. However, studies on the quality changes of LF powders after long-term storage are lacking (e.g. >5 years), which limits consumers’ understanding of the safety and effectiveness of FL powders stored for a long period. Previous studies have reported that temperature, hydration, pH and other environmental stresses during storage could affect the stability of protein powders, thus weakening the interaction between polysaccharides and other proteins, which may lead to protein denaturation, nutritional value reduction and loss of functions (Ward et al., 2005).

This study aims to compare the differences in various functional properties between LF powders stored for 9 years and commercial LF powders available on the market. Physicochemical characteristics such as protein and moisture contents, colour profiles and water solubility of LF powders were measured. The bacteriostatic, antioxidant and iron-binding functions of the LF powders were also determined. Overall, this study reveals the long-term storage performance of LF powders, providing a theoretical basis for future food applications such as for long voyages and aerospace flights.

## 2 Materials and methods

### 2.1 Materials

Old bLF powders (LF_2016_) were produced by freeze drying fresh bLF solution (16 %–18 %, w/w) from Tatura Milk Industries Ltd. LF_2024_ is the LF_2016_ sample stored at 4 °C for 9 years. Commercial LF powders were obtained from the following: (1) Warrnambool Cheese and Butter Ltd. (Victoria, Australia), freeze-dried; (2) The Tatua Co-operative Dairy Company (Morrinsville, New Zealand), LF004-P08-09, freeze-dried; (3) Hilmar Cheese Company Inc. (California, USA), Hilmar™ 1000, freeze-dried; (4) Mercurius Production GmbH (Frankfurt am Main, Germany), MIG-95 CHN SD FNE, spray-dried; (5) FrieslandCampina Nederland B.V. (LE Amersfoort, the Netherlands), spray-dried; (6) Tatura Milk Industries Ltd. (Subsidiary of Bega Cheese, Victoria, Australia), spray dried; and (7) Feihe Dairy Co., Ltd. (Heilongjiang, China), freeze-dried. Recombinant human LF was donated by (8) Nanjing Bestzyme Bio-Engineering Co., Ltd. (Jiangsu, China), freeze-dried. All the LF powders were stored in the refrigerator at 4 °C and 40 % RH. The Pierce™ BCA Protein Assay Kit was acquired from Thermo Scientific (Victoria, Australia). Mini-PROTEAN® TGX™ gels, WesternC™ blotting standards, Laemmli sample buffer, Tris/glycine/SDS buffer and mercaptoethanol were purchased from Bio-Rad. Lysozyme from chicken egg white, 2,2-diphenyl-1-picrylhydrazyl (DPPH) and 2,2′-azino-bis (3-ethylbenzothiazoline-6-sulphonic acid) (ABTS) were purchased from Shanghai Macklin Biochemical Technology Co., Ltd. (Shanghai, China). The standard iron solution was purchased from Beijing Coastal Hongmeng Standard Material Technology Co., Ltd. (Shanghai, China). All chemicals listed above were of analytical grade and were used as received.

### 2.2 Preparation and storage of bLF powders

bLF powders (LF_2016_) were prepared using a laboratory-scale freeze dryer (FreeZone™, Labconco Inc., USA). The freeze-drying process consisted of 12 h primary drying (0 °C) and 6 h secondary drying (20 °C). More details were provided in a previously reported study (B. Wang et al., 2017a). The bLF powders were sealed in high borosilicate glass bottle and kept at a constant cool temperature (4 °C) and regular humidity (40 % RH) for 9 years. The relative humidity of 40 % is often used as one of the standard testing conditions in laboratories according to the international standards (ISO and ASTM). During the long-term storage, the humidity in the refrigerator and inside the packaging bags were monitored using a high-precision hygrometers (Deli 8848, China).

### 2.3 Measurement of moisture and protein contents

The moisture content of the bLF powders was determined using AOAC 930.15 (Panda et al., 2022). Briefly, 100–120 mg of bLF powders was heated in an oven to 105 °C for 6 h. The moisture content was quantified by calculating the percentage of weight changes.

The protein content of the bLF powders was measured using the bicinchoninic acid (BCA) assay method (B. Wang et al., 2017a). The protein standard (BSA) was diluted to a final concentration in the range of 0–2000 μg/mL. The Pierce™ BCA Protein Assay Kit (Thermo Scientific, Victoria, Australia) was used to measure the protein content. Pierce™ BCA Protein Assay Reagent A (50 mL) and Pierce™ BCA Protein Assay Reagent B (1 mL) were mixed to prepare the working liquid. The bLF sample was dissolved in ultra-pure water to a concentration of 1 mg/mL. Then, 100 μL of bLF solution was mixed with 2 mL of working liquid, and the mixture was incubated at 37 °C for 30 min. A UV spectrophotometer (UV759S, Shanghai Lengguang Scientific, China) was used to measure the absorbance value at 562 nm with ultrapure water as the blank.

The protein content of bLF powders were also determined using a Kjeldahl nitrogen determination apparatus (Hanon K9860, China) following the method described by AOAC 960.52 (Ma et al., 2021). The protein coefficient of LF was 6.38. Results of the Kjeldahl nitrogen measurement are provided in the supplementary data.

### 2.4 Determination of molecular weight

The molecular mass of LF was determined using sodium dodecyl sulfate–polyacrylamide gel electrophoresis (SDS–PAGE) analysis (F. Wang et al., 2020). Briefly, bLF powders were dissolved in ultra-pure water to achieve a concentration of 2 mg/mL. Subsequently, 100 μL of the supernatant was combined with an equivalent volume of SDS sample buffer, which consisted of 95 % Laemmli buffer and 5 % β-mercaptoethanol. After thorough mixing, the bLF samples were heated at 95 °C for 5 min. An 8 μL aliquot of the heated mixture was loaded into the sample wells of the precast gel, and 15 μL of the marker was loaded. Electrophoresis was performed using the Bio-Rad Mini-PROTEAN Tetra vertical electrophoresis system. The direct current voltage was set to 100 V by the electrophoresis device, and electrophoresis was run for approximately 75 min. After electrophoresis, the gel was taken out and dyed with 0.1 % Coomassie Brilliant Blue R-250. Subsequently, the gel was decoloured with a mixture consisting of 40 % formic acid, 10 % acetic acid and 50 % ultra-pure water. The electrophoresis outcomes were recorded using a digital camera, and LF purity was assessed using Image J analysis software. LF purity was determined using the percentage of the LF band's grey level relative to the total grey level of the gel.

### 2.5 Measurement of colour and water solubility

The colour of the bLF powders was analysed using a colorimeter (CR-400, Konica Minolta, Japan) (Feng et al., 2021). A standard white plate was used for calibration.

The water solubility of the bLF powders was measured according to the method (F. Wang et al., 2020). Briefly, 100 mg of bLF powders was dissolved in a centrifuge tube filled with 1 mL of ultra-pure water. The bLF solution was agitated at 400 rpm for 30 min, followed by centrifugal separation at 11800×*g* for 50 min using a small centrifuge (Eppendorf AG, 5424FR173085, USA). The supernatant obtained from centrifugation was discarded, and the remaining sediment was desiccated in an oven preheated to 105 °C for 4 h. Water solubility was calculated using Eq. (1):

(1) $$\text{Solubility}\;\left(\%\right)=\left[1-\left(W_{before-}W_{after}\right)/\;W_{sample}\right]\times 100\%$$

where $W_{sample}$ is the weight of the bLF powders (mg), $W_{before\;}$ is the weight of the bLF solution before drying (mg) and $W_{after}$ is the weight of the bLF solution after drying (mg).

### 2.6 Measurement of particle size and zeta potential

The particle size and zeta potential of the bLF samples were assessed using a laser particle size potentiometer (Marvin Zetasizer Lab, UK) (Liang et al., 2021). For the particle size analysis, 2 mg/mL bLF solution was prepared and transferred to a Malvern cuvette (DTS0012). For the determination of zeta potential, a solution of bLF at a concentration of 2 mg/mL was prepared and transferred to a Malvern zeta potential sample cell (DTS1070). The measurement was conducted at 25 °C with an equilibrium time of 120 s.

### 2.7 Monitoring surface morphology

The morphological features of the bLF powders were examined using a scanning electron microscope (SEM, Quanta FEG 250, Japan) without gold sputtering (Wen et al., 2020). The SEM was operated with an acceleration voltage of 15 kV.

### 2.8 Determination of iron content and iron-binding ability

The iron content of the LF samples was measured using an inductively coupled plasma-mass spectrometer (ICP-MS, 2030LF, Japan). Mineralisation was performed using a microwave digestion system (TANK ECO, China).

The iron-binding ability was determined by quantifying the maximum amount of iron that can bind to bLF (Xia et al., 2023). Briefly, bLF powders were dissolved in ultra-pure water to prepare 10 mg/mL bLF solution. The bLF solution was adjusted to pH 6. Excessive iron (10 mM ferric trichloride and 10 mM hyponitrotriacetic acid) was mixed with the sample for 1 h. Next, the solution was dialysed using ultra-pure water for 48 h, with the ultra-pure water being changed every 6 h. The dialysed samples were resuspended with phosphate-buffered saline (PBS) and passed through luer syringe filters. The samples were microwave digested and subjected to ICP-MS. The iron-binding ability was calculated using Eq. (2):

(2) $$\text{Iron}-\text{binding}\;\text{ability}\;\left(\text{mg}/\text{mg}\right)={{Iron}_{saturation}-Iron}_{LF}$$

where ${Iron}_{LF}$ is the mass of iron in the bLF samples and ${Iron}_{saturation}$ is the mass of iron in the bLF solution after reacting with excessive iron.

### 2.9 Determination of denaturation profiles

The temperature of denaturation and the extent of LF samples were determined using a differential scanning calorimetry apparatus (DSC, Mettler-Toledo DSC 3, Switzerland) (Zink et al., 2024). For this purpose, 60 μL of the bLF solution (20 %, w/v) was transferred to an aluminium crucible and sealed hermetically. An identical volume of ultra-pure water served as the control. The specimens were warmed from 30 °C to 96 °C at a rate of 5 °C/min. The nitrogen flow rate was set to 20 mL/s. The enthalpy changes in the ranges of 60°C–70 °C and 88°C–96 °C were used to analyse the denaturation temperature and degree of the bLF samples.

### 2.10 Thermal analysis

The thermal stability of the LF samples was determined using thermogravimetric analysis (TGA2, Mettler-Toledo, Switzerland) (Feng et al., 2021). The samples (9 mg) were placed in a ceramic crucible, N_2_ was selected as the protective gas for thermogravimetric analysis (TGA) measurement and the ceramic crucible was subjected to a temperature increase from 25 °C to 600 °C at a speed of 10 °C/min. The temperature range was divided into two regions (Region I: 25–150 °C; Region II: 150–600 °C), each corresponding to the maximum loss of free and bound water in the LF sample, as well as the maximum degradation rates. The mass loss in these two temperature regions was determined by the derivative thermogravimetric curves.

### 2.11 Determination of functional groups

FTIR spectra were collected using a Fourier infrared spectrometer (Nicolet iS50, USA) (Wen et al., 2020). All spectra were the average of 32 scans in the range 4000–500 cm^−1^, with a resolution of 4 cm^−1^.

### 2.12 Determination of crystal structure

The amorphous/crystalline properties of the LF powder were measured using an X-ray diffractometer (SmartLab, Rigaku, Japan) (Yu et al., 2020). The diffraction scanning range (2θ) was 5°–55°, the scanning speed was 4°/min, the step size was fixed at 0.02° and the ratio of the sum of the areas of each effective peak to the total AUC was calculated as relative crystallinity.

### 2.13 Determination of structural changes

The alteration of the secondary structural features of bLF was determined using circular dichroism (CD) spectroscopy (J-815, JASCO, Japan) (F. Wang et al., 2020). For this purpose, bLF powders were dissolved in ultra-pure water to prepare 0.04 mg/mL bLF solutions. During the CD experiment, the temperature was adjusted to 25 °C. The spectrum was analysed from 190 to 260 nm at a scan rate of 50 nm/min and a bandwidth of 1 nm.

### 2.14 Determination of antioxidant capacity

(1) DPPH radical scavenging capacity assay

The antioxidant capacity of bLF was determined by comparing the DPPH and ABTS scavenging abilities of different samples (Wei et al., 2025). The DPPH solution (0.1 mmol/L, dissolved in 95 % ethanol) was mixed with an equal volume of bLF solutions (5, 10, 15 and 20 mg/mL, dissolved in ultra-pure water). The mixture was vortexed for 5 s and then incubated at 25 °C in the dark for 30 min. Then, the reacted solution was transferred to a quartz cuvette to measure its absorbance at 517 nm. A blank sample was created by combining the LF solution with a matching volume of 95 % ethyl alcohol. A reference sample was made by blending DPPH solution with the same volume of 95 % ethyl alcohol. The calculation formula for the inhibition rate of DPPH free radicals is shown in Eq. (3):

(3) $$\text{DPPH}\;\text{Free}\;\text{Radical}\;\text{Inhibition}\;\text{Rate}\;\left(\%\right)=\left(A_{sample}-A_{blank}\right)/A_{control}\times 100\%$$

where $A_{sample}$ is the absorbance of bLF + DPPH, $A_{blank}$ is the absorbance of bLF + ethanol and $A_{control}$ is the absorbance of DPPH + ethanol.

(2) ABTS radical scavenging capacity assay

The ABTS stock solution was prepared by blending 7 mM ABTS with 2.45 mM potassium persulphate and allowing the mixture to react at ambient temperature for 16 h in the dark (Wei et al., 2025). The ABTS working liquid was prepared by diluting the stock solution with PBS buffer of pH 7.4 so that its absorbance at 734 nm was 0.700 ± 0.001. The ABTS working fluid was set as the control (Ac). LF aqueous solutions with concentration of 5, 10, 15 and 20 mg/mL were configured. The absorbance at 734 nm was measured after the reaction of 20 μL of the sample mixed with 3 mL of the ABTS working solution for 6 min (As). The calculation formula for the inhibition rate of ABTS free radicals is shown in Eq. (4):

(4) $$\text{ABTS}\;\text{Free}\;\text{Radical}\;\text{Inhibition}\;\text{Rate}\;\left(\%\right)=\left(A_{c}-A_{s}\right)/\;A_{c}\times 100\%$$

where $A_{c}$ is the absorbance of the ABTS working fluid and $A_{s}$ is the absorbance of the bLF + ABTS working fluid.

### 2.15 Determination of antibacterial activity

*Escherichia coli*, *Staphylococcus aureus* and *Salmonella enteritidis* were selected as the target bacteria (L. Lin et al., 2019). The antibacterial effects of bLF against the above three bacteria were analysed using the plate counting method (Lingle et al., 2022).

Briefly, 100 μL of each bacterial species was added to 1 mL of aseptic LB broth and grown at 37 °C over a period of 4 h. Then, *E. coli*, *S. aureus* and *S. enteritidis* were streaked separately on MacConkey, TSA and SS agar plates, respectively. Individual colonies were selected from the agar plates, transferred into 1 mL of sterile LB broth and cultured at 37 °C on a shaker for 4 h until the logarithmic phase.

The bLF solution (64 mg/mL) was prepared using sterile water and filtered through a 0.22-μm sterile filter. Then, 50 μL of bacterial suspension, 2 mL of bLF solution and 5 mL of liquid medium were inoculated together in a sterile shaking tube and cultured at 37 °C on a shaker for 12 h. The shaking speed was 120 rpm. The cultured bacterial suspension was diluted to a gradient of 10^6^ and spread on plates. The plates were placed in a 37 °C incubator for 12–18 h. After incubation, the plates were counted.

### 2.16 Haemolysis test

Haemolysis degree was measured to assess the compatibility of LF with red blood cells. Defibrinated sheep blood was used to detect the hemolysis rate of LF with red blood cells (M. Li et al., 2022). First, the defibrillated sheep blood was centrifuged at 1450×*g* for 10 min, the upper layer was disposed and the settled red blood cells were cleaned thrice using normal saline to create a diluted blood sample. LF_2024_ samples with different concentrations (1, 5, 10, 15 and 20 mg/mL) were immersed in normal saline (10 mL) as the experimental group. The negative and positive controls were 10 mL of normal saline and 10 mL of Triton X-100 (Triton X-100, 1 %), respectively. Diluted blood (200 μL) was added to each group and incubated at 37 °C for 4 h. Following incubation, the supernatant was obtained via centrifugation at 1450×*g* for 10 min and then detected using an ultraviolet spectrophotometer (UV759S, Shanghai Lengguang Scientific, China) at 545 nm. The formula for calculating the haemolysis rate is shown in Eq. (5):

(5) Haemolysisrate(%)=(As−An)/(Ap−An)×100%

where As is the light absorption value of the test group, An is the absorption value of the negative control and Ap is the absorption value of the positive control.

### 2.17 Statistical analysis

Three replicate tests for each experimental setup were conducted to evaluate variability, with the outcomes expressed as average ± standard deviation (SD). Statistical significance was examined using one-way analysis of variance via SPSS Statistics 27 (IBM Corporation, New York, USA). The comparative significance between various samples was analysed, with p values of <0.05 indicating statistical significance.

## 3 Results and discussion

### 3.1 Physicochemical properties

#### 3.1.1 Moisture, protein, solubility and colour of LF powders

The physicochemical properties of the LF powders are presented in Table 1. The old bLF powders (LF_2016_) produced from a bench scale freeze dryer had a low moisture content (2.7 %), which is similar to that of the commercial LF powders (2.8 %–5.3 %). After 9 years of storage, the bLF powders (LF_2024_) absorbed considerable amount of moisture from the environment, leading to the increase in moisture content (8.8 %). This was also evitable during the storage of many other food powders (Norwood et al., 2019; Urgu-Öztürk et al., 2021). For example, after 12 months of storage, the water content of cheese powder increased by 0.75 %, which may be due to the increased water activity. According to research (Shen et al., 2024), during storage, the increase in water activity inside the packaging is directly related to the barrier performance of the packaging material to water vapour. At 75 % relative humidity, water is more easily permeated into the packaging, thereby increasing the water activity inside the powder. During storage, amorphous lactose promotes the powder's absorption of water. This is because powders containing a high proportion of amorphous lactose have a strong hygroscopicity, and this property can be further exacerbated by the presence of water-absorbing proteins. Interestingly, the protein content and water solubility of LF_2024_ only showed negligible changes after the long-term storage. LF_2024_ still had 90.9 % protein and 93.0 % water solubility, falling in the range of commercial LF powders. However, the BCA method may be subject to interference from certain reducing substances in the sample or other compounds that can react with the BCA reagent, which may lead to inaccurate measurement results. The Kjeldahl method, on the other hand, reduces these interferences through the digestion process. Kjeldahl nitrogen determination results are included in the supplementary data. The colour of LF_2024_ was brighter, redder and yellower than LF_2016_. The increase in L∗ value (lightness) might be due to the water invasion of LF_2024_. The low diffusivity of water leads to internal evaporation and periodic escape of water, resulting in porosity, where water enters to fill the pore gaps between bLF particles, resulting in light scattering (B. Wang et al., 2017a). The increase in a∗ value and b∗ value might be attributed to the more compact structure of LF_2024_. It has also been reported that during long-term storage, the voids in powders were reduced (Burgain et al., 2016).

**Table 1:** Physicochemical properties of different LF powders.

Samples	Moisture content (%)	Protein content (%)	Water solubility (%)	Colour		
				L∗	a∗	b∗
**LF_2016_**	2.7 ± 0.5^a^	92.1 ± 0.2^b^	93.5 ± 0.1^abc^	72.9 ± 1.6^ab^	8.8 ± 0.6^c^	15.7 ± 0.8^cde^
**LF_2024_**	8.8 ± 0.8^b^	90.9 ± 2.7^c^	93.0 ± 1.7^abc^	72.9 ± 0.1^bcd^	10.9 ± 0.2^de^	16.8 ± 0.2^de^
Brand1	2.8 ± 1.7^a^	89.9 ± 1.2^d^	93.5 ± 0.9^abc^	68.1 ± 0.6^a^	11.4 ± 0.7^e^	17.2 ± 0.5^e^
Brand2	3.2 ± 1.9^a^	89.0 ± 1.7^d^	91.9 ± 2.1^a^	72.9 ± 0.8^bc^	9.9 ± 0.2^cde^	16.5 ± 0.3^de^
Brand3	2.6 ± 1.9^a^	92.2 ± 2.4^ef^	92.7 ± 0.7^ab^	77.0 ± 2.9^def^	7.2 ± 0.1^b^	13.0 ± 0.2^ab^
Brand4	3.5 ± 1.5^a^	87.5 ± 2.8^d^	95.3 ± 0.3^c^	73.2 ± 0.5^bcde^	9.5 ± 0.7^cd^	15.9 ± 0.4^cde^
Brand5	4.1 ± 1.6^a^	86.7 ± 0.5^a^	94.0 ± 1.4^abc^	79.3 ± 1.2^f^	6.6 ± 1.2^ab^	14.3 ± 0.6^bc^
Brand6	5.3 ± 0.3^a^	91.6 ± 1.6^c^	93.3 ± 1.3^abc^	77.2 ± 1.7^ef^	7.6 ± 1.6^bc^	14.6 ± 1.1^cd^
Brand7	3.0 ± 1.4^a^	91.9 ± 2.3^ef^	92.9 ± 1.9^ab^	84.7 ± 0.8^g^	6.6 ± 0.4^a^	14.9 ± 0.4^cd^
Brand8	4.0 ± 1.3^a^	91.3 ± 0.6^f^	94.5 ± 0.5^bc^	76.0 ± 1.8^cdef^	6.9 ± 0.3^ab^	12.4 ± 0.2^a^

Data of LF_2016_ were acquired from a previous study published 9 years ago (B. Wang et al., 2017a). Mean values with different lower case letters in superscript at the same column are significantly different (p < 0.05).

#### 3.1.2 Hydrodynamic size and zeta potential of LF

**Fig. 1:**
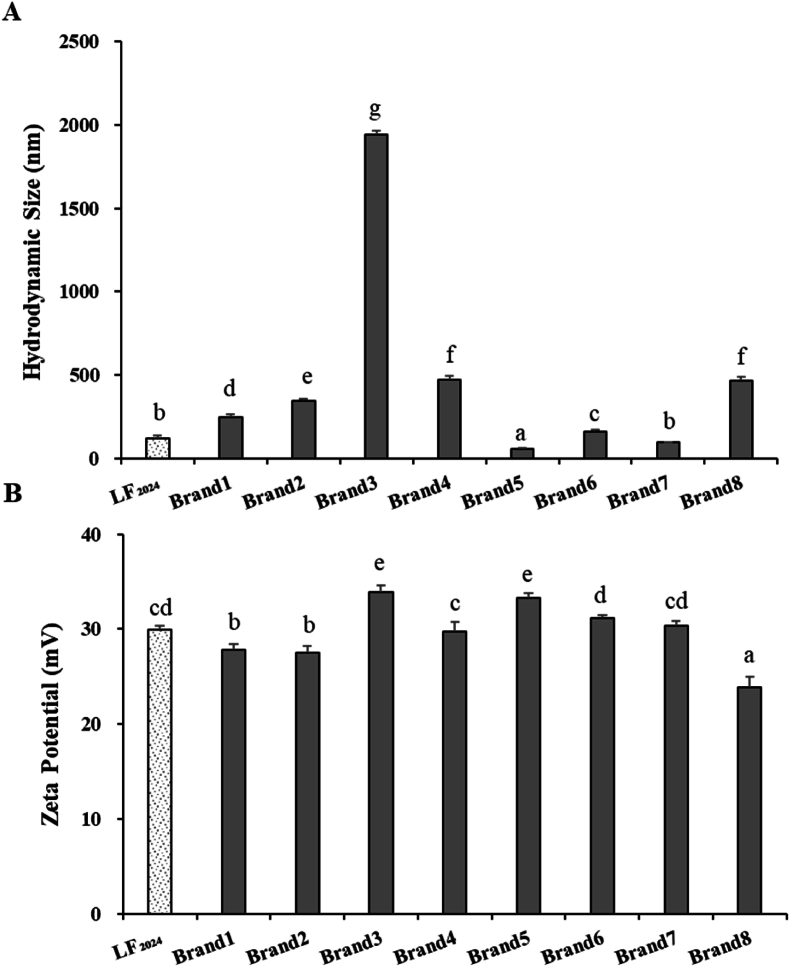
A) Hydrodynamic size and B) zeta potential of LF samples. LF_2024_ is the bLF sample stored at 4 °C for 9 years. Brands 1–8 are commercial LF powders.

The hydrodynamic size and zeta potential of LF are presented in Fig. 1. The hydrated particle size and zeta potential of the LF powders were significantly different (p < 0.05; Fig. 1A and B). The bLF powder stored for 9 years (LF_2024_) had a smaller hydrated particle size compared with the commercial LF powder when both powders were subjected to the same drying process (Brand1, Brand2, Brand3, Brand7 and Brand8), probably because the long-term stored bLF powder underwent a reversible structural development during storage. Thus, the exposure of the non-covalent binding sites on the protein surface was reduced. It was also easier to disperse to the relevant solution system, which was conducive to exerting its physiological activity. It is interesting to note that Brand 3 showed much higher particle size than all the other samples. The reason for this phenomenon is not very clear. It is suspected that some freeze-drying parameters such as freezing rates, vacuum conditions and heating rate could affect the formation of crystals or promote the agglomeration between particles. These might result in higher hydrodynamic particle size (Z. Wang et al., 2025). In addition, the particle size of the bLF powder in the water phase was closely related to its water solubility. As noted in Table 1, the lower solubility of LF_2024_ compared with that of LF_2016_ led to a decrease in its actual solubility in water, resulting in a smaller measured hydrated particle size. Results showed that the molecular structure of the LF_2024_ powder remained stable with increasing storage time.

The bLF powder is a cationic protein and is positively charged. As shown in Fig. 1B, the zeta potential of LF_2024_ (29.9 mV) fall into the range of zeta potential values of commercial LF powders (25–33 mV). The zeta potential of proteins can serve as an indicator for assessing particle interactions subsequent to protein hydrolysis. High-zeta-potential LF usually exhibits good dispersion stability because its charged surface prevents its aggregation and precipitation in solution (Voswinkel et al., 2016). Hence, an increase in the absolute value of the zeta potential is inversely proportional to the aggregation degree in the sample system, leading to enhanced stability. The minimal change in zeta potential and particle size of LF_2024_ may be due to the change in protein structure. At low-temperature (4 °C) storage, the molecular diffusion rate slows down, and the collision frequency between small droplets and particles reduces; thus, merging is not easy for the formation of larger particles (S. Wang et al., 2022). Because LF has two iron-binding sites, the binding of these sites to iron ions can affect the structure and stability of the protein. During storage, LF_2024_ is placed in a low-temperature (4 °C), low-humidity and low-oxygen environment to help in maintaining protein stability and preventing the release of iron ions. Research indicates that the Fe^3+^ ions in LF are situated in its deep fissures. In the absence of iron ions, these fissures can be widened by manipulating the protein's structure. However, with increasing concentration of iron ions, more Fe^3+^ ions attach to LF, infiltrate the cleavages between protein layers and induce the fissures to seal, thereby leading to a more compact molecular configuration of the protein (Xie et al., 2024). With an extension of the storage time, the particle size of LF_2024_ decreases and the zeta potential increases. These results indicate that the bLF powder (LF_2024_) exhibits good stability and solubility.

#### 3.1.3 Protein integrity and protein molecular weight

**Fig. 2:**
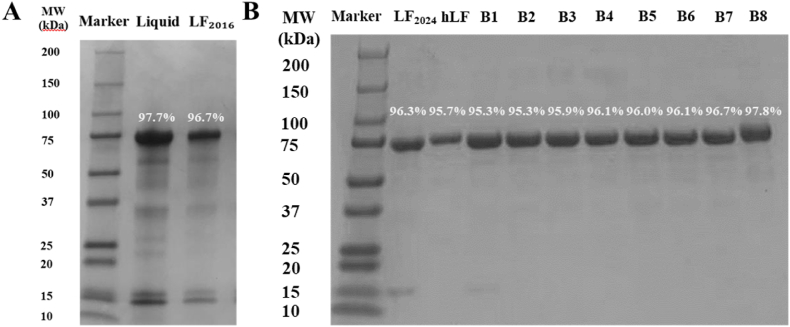
Protein molecular weight patterns of A) LF_2016_, B) LF_2024_, hLF and Brand 1–Brand 8. LF_2016_ and LF_2024_ are the bLF powders produced in 2016 and the same sample stored at 4 °C until 2024. hLF is human LF extracted from human milk. B1–B8 are commercial LF powders. The white numbers above the bands indicate the corresponding band intensity. Data of LF_2016_ were acquired from a previous study published 9 years ago (B. Wang et al., 2017a).

The molecular weight distribution of the bLF powders obtained using the reductive SDS–PAGE experiment is shown in Fig. 2. The main band of all LF powders appeared at 75 kDa, and the molecular weight spectrum was consistent with the results of previous studies (Bokkhim et al., 2016; B. Wang et al., 2017a). The bLF powder stored for a long period (LF_2024_) showed no significant change in the intensity of the 75 kDa band compared with the 9-year-old bLF powder (LF_2016_; Fig. 2A and B). The weak band at 15 kDa might be α-opal, β-lactoglobulin and other miscellaneous proteins that have not been completely removed during bLF extraction. This is consistent with reported literature, which mentions that LF is prone to degradation under alkaline conditions and heat treatment but stable at regular storage conditions (Osel et al., 2021). Consequently, the purity of LF_2024_ was 96.3 %, similar to that of LF_2016_ (96.7 %) and the commercial LF powders (95.3 %–97.8 %).

#### 3.1.4 Surface morphology of LF powders

**Fig. 3:**
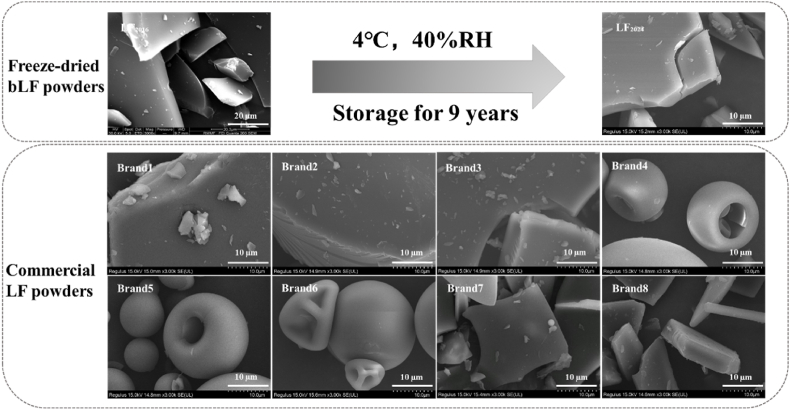
SEM images of LF powders. LF_2016_ is the LF powder obtained in 2016, and LF_2024_ is the LF_2016_ sample stored at 4 °C for 9 years. Brands 1–8 are commercial LF powders. Data of LF_2016_ were acquired from a previous study published 9 years ago (B. Wang et al., 2017a).

The surface morphology of the bLF powders was monitored using SEM (Fig. 3). After 9 years of storage, the surface morphology of the bLF powder (LF_2024_) showed adhesion and partial fragmentation, which might be caused by an excessive water content (Table 1). It might be due to shear forces caused by high moisture, which undermined the interchain cross-links in proteins, such as disulphide bridges, hydrogen bonds and van der Waals attractions (Jin et al., 2021). LF_2024_ exhibits a similar surface morphology as that of LF powder under the same drying process, both presenting a relatively smooth surface. It has been reported that surface morphological changes are related to hydrophobic interactions on protein surfaces. However, the precise underlying mechanism remains unclear.

### 3.2 Structure feature

#### 3.2.1 Fourier transform infrared (FTIR)

**Fig. 4:**
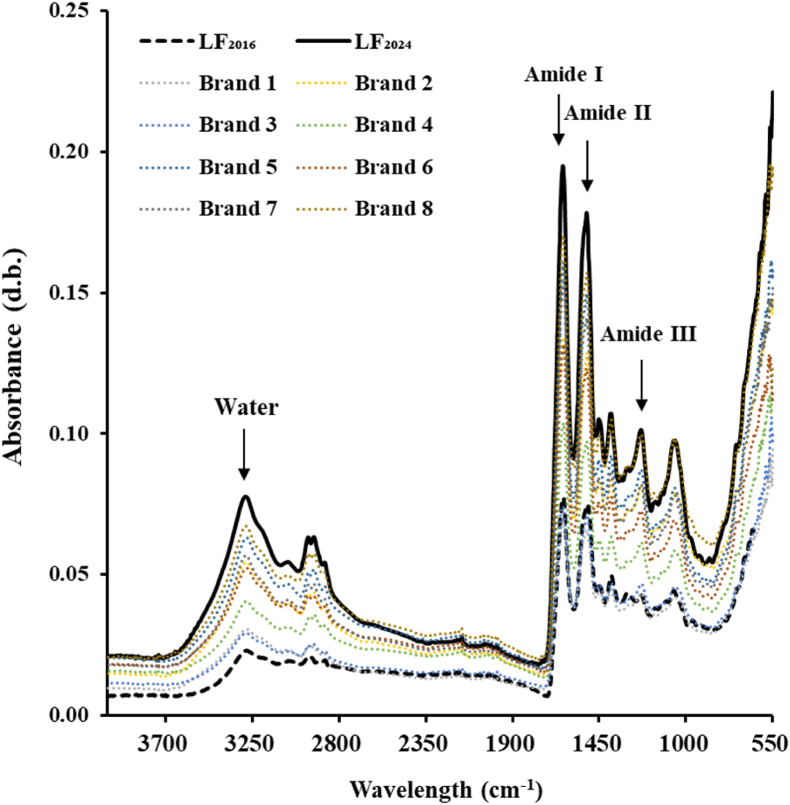
FTIR spectra of different LF powders. LF_2016_ is the LF powder obtained in 2016, and LF_2024_ is the LF_2016_ sample stored at 4 °C for 9 years. Brands 1–8 are commercial LF powders. Data of LF_2016_ were acquired from a previous study published 9 years ago (B. Wang et al., 2017a).

FTIR is one of the main methods for the effective analysis of protein functional group changes. As shown in Fig. 4, FTIR spectroscopy reveals characteristic absorption peaks for proteins in the amide I, II and III regions. Among these, the amide I and amide II regions are frequently employed for both qualitative and quantitative assessments of protein secondary structure (Wei et al., 2025). The amide I band (1600–1700 cm^−1^) mainly represents the plane stretching of C=O, hydrogen bonding and COO-coupling, and the amide II band (1500–1600 cm^−1^) mainly reflects N–H bending and C–N stretching (Wen et al., 2020). As shown in Fig. 4, all LF powders had similar peaks in the FTIR spectrum. LF_2024_ powders had a strong water absorption peak at 3200–3500 cm^−1^, which was due to O–H tensile vibration. This also confirms that after 9 years of storage, bLF powders have absorbed a large amount of water from the environment, which is consistent with the increase in moisture content (Table 1). Compared with eight commercially available LF powders, LF_2024_ did not have a significant red-shift or blue-shift, indicating that its structure was relatively stable.

#### 3.2.2 X-ray diffraction

**Fig. 5:**
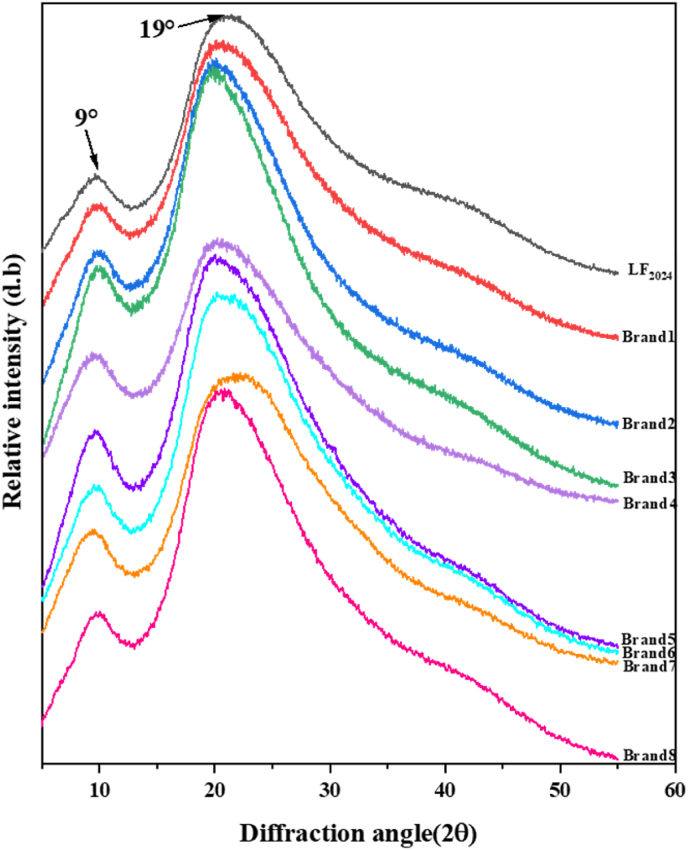
X-ray diffraction of different LF powders. LF_2024_ is the LF_2016_ sample stored at 4 °C for 9 years. Brands 1–8 are commercial LF powders. (Note: LF_2016_ is not included in this figure for comparison because of the instrument differences).

The crystal structure of the bLF powders was studied using X-ray diffraction (XRD) spectroscopy. As shown in Fig. 5, the XRD of bLF exhibited a typical amorphous structure, where two prominent peaks were observed at 2θ = 9° and 19°. The peaks that appeared at 2θ = 9° corresponded primarily to an α helix structure, and the peaks shown at 19° corresponded to a β-sheet structure (Feroz et al., 2022). LF_2024_ and commercial LF powders have similar XRD profiles and wider crystallisation peaks, indicating amorphous properties. Results showed that the LF powder was not easy to crystallise during long-term storage at 4 °C and 40 % RH.

#### 3.2.3 CD

**Fig. 6:**
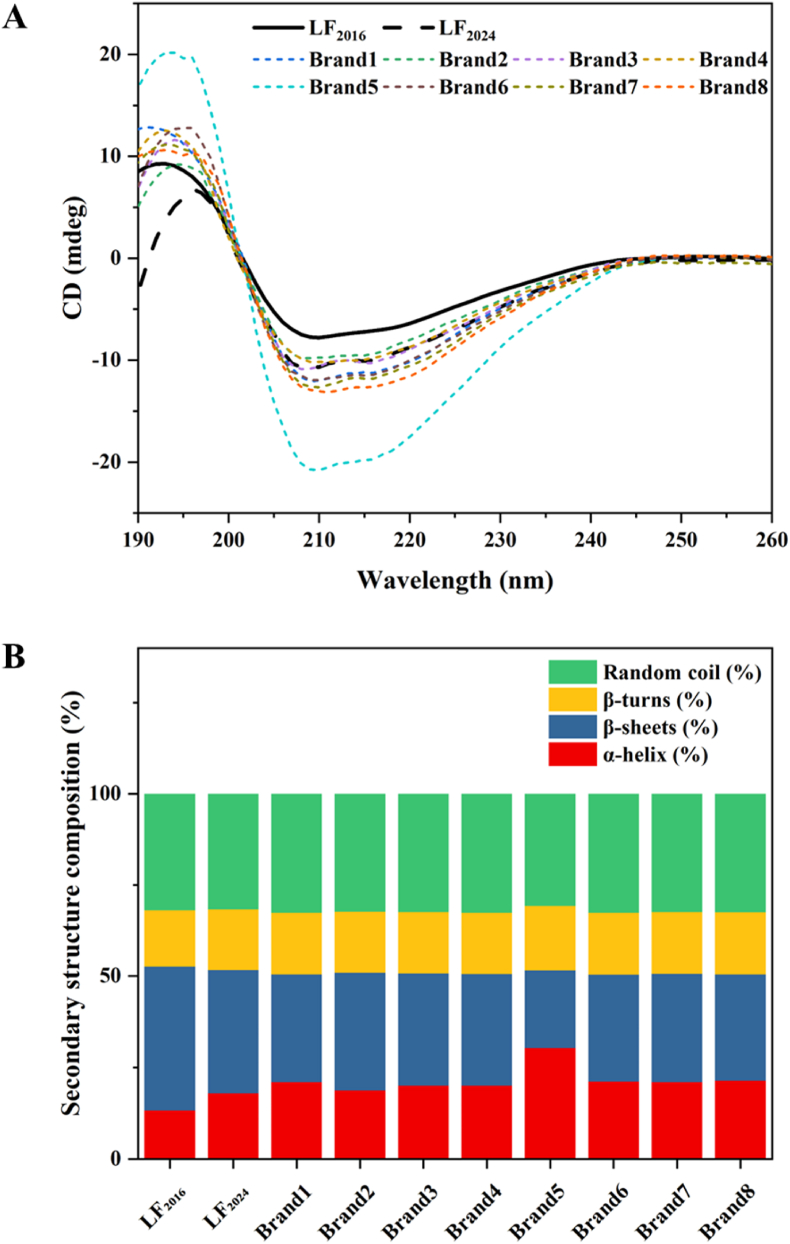
A) CD spectrum and B) secondary structure composition of LF samples. LF_2016_ is the LF powder obtained in 2016, and LF_2024_ is the LF_2016_ sample stored at 4 °C for 9 years. Brand 1–Brand 8 are commercial LF powders. Data of LF_2016_ were acquired from a previous study published 9 years ago (B. Wang et al., 2017a).

The CD spectrum of the bLF powders is shown in Fig. 6A. The variation in ellipticity indicated the degree of protein conformational change caused by different processing processes. The spectral curves of all LF powders exhibited negative peaks near 208 nm, corresponding to a typical α-helix structure, which is consistent with previous studies (Q. Li et al., 2019). Similarly, the LF powder samples showed a positive peak near 195 nm, corresponding to a typical β-folding structure. After 9 years of storage at 4 °C, the secondary structure of the bLF powders changed slightly, with α-helix and β-turns increased and the content of β-sheets and random coil decreased (Fig. 6B). In general, the increase in α helix and β corners contributes to the formation of a stable and ordered protein secondary structure. However, the alpha helix did not decrease during storage, which might be because low temperatures reduced the breaking of the hydrogen bonds, reduced the solubility and reactivity of oxygen and reduced the damage of oxidation to the protein structure. Low temperatures can reduce non-specific interactions between proteins, thereby preventing protein aggregation and precipitation (Majid and Khan, 2023). Significantly, in this context, lower alpha-helices in proteolytic solutions may contribute to enhanced antioxidant activity. Therefore, with an extension of storage time, the secondary structure of LF_2024_ remained stable. Meanwhile, the secondary structure of LF_2024_ was similar to that of commercial LF powders.

#### 3.2.4 Extent of denaturatio of bLF powders

**Fig. 7:**
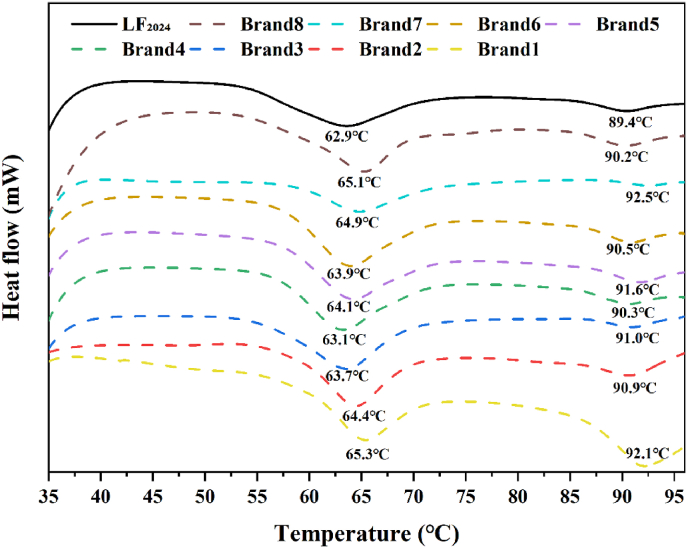
DSC thermograms. LF_2024_ is the LF_2016_ sample stored at 4 °C for 9 years. Brand 1–Brand 8 are commercially available LF powders. (Note: LF_2016_ is not included in this figure for comparison because of the instrument differences).

The denaturation temperatures of the bLF powders are presented in Fig. 7. The LF_2024_ sample showed two denaturation peaks, consistent with literature (Goulding et al., 2021). The first peak occurred at approximately 63.5 °C; the second, at 90.1 °C (Table 2). It has been reported that the thermal denaturation peaks of iron-depleted bLF and iron-saturated bLF are approximately 58 °C and 89 °C, respectively (Goulding et al., 2021). The enthalpy (ΔH) of the first peak of LF_2024_ was −0.8 J/g, and its absolute value was significantly (P < 0.05) higher than that of the second peak (−0.1 J/g), which corresponded to the N and C lobes of bLF, respectively. The difference in iron saturation and structure density between the N and C segments in bLF might be the reason for their different thermal stability values (T. Lin et al., 2022). The ΔH of the bLF powder stored for 9 years (LF_2024_) was slightly lower than that of commercial LF powder but still in the acceptable range, possibly for reasons related to its higher moisture content, resulting in conformational instability and low thermal stability. In addition, it has been shown that the enthalpy value (ΔH) indicates the degree to which a protein undergoes structural change during heating, reflecting the proportion of ordered structures (Y. Wang et al., 2024). As the denaturation temperature increases, the protein's tertiary structure becomes more tightly packed, leading to enhanced thermal stability. A larger ΔH indicates that the protein is more compact and conformationally stable, and a smaller ΔH indicates that the protein is more extended with fewer hydrogen bonds and is conformationally unstable. It has been reported that the thermal stability of bLF increases with increasing iron saturation levels (Bokkhim et al., 2013). This effect is further discussed in Section 3.3.

**Table 2:** Denaturation temperatures of LF.

Samples	Thermal parameters of LF			
	T_onset_ (°C)	T_peak_ (°C)	T_endset_ (°C)	ΔH (J/g)
Corresponding to iron-depleted bLF				
LF_2024_	57.5 ± 0.3^a^	63.5 ± 0.5^a^	69.5 ± 0.5^ab^	0.8 ± 0.2^a^
Brand1	59.9 ± 0.2^ab^	65.3 ± 0.3^bc^	71.0 ± 0.3^b^	2.0 ± 0.6^e^
Brand2	58.7 ± 2.3^ab^	64.6 ± 0.8^abc^	70.0 ± 0.6^ab^	1.4 ± 0.8^c^
Brand3	59.0 ± 1.4^ab^	64.2 ± 1.1^ab^	68.8 ± 1.7^a^	1.3 ± 0.6^c^
Brand4	58.9 ± 0.2^ab^	64.2 ± 0.4^ab^	69.6 ± 0.4^ab^	1.7 ± 0.9^d^
Brand5	59.9 ± 0.1^ab^	65.8 ± 0.3^c^	70.5 ± 0.5^b^	0.7 ± 1.2^a^
Brand6	59.3 ± 0.1^ab^	64.9 ± 0.5^abc^	70.5 ± 0.6^ab^	1.1 ± 0.5^b^
Brand7	61.5 ± 3.8^b^	65.3 ± 1.6^bc^	70.0 ± 1.4^ab^	1.2 ± 0.2^b^
Brand8	58.0 ± 0.1^a^	65.2 ± 0.1^bc^	70.1 ± 0.4^ab^	1.8 ± 0.4^d^
Corresponding to iron-saturated LF				
LF_2024_	87.0 ± 0.9^bc^	90.1 ± 0.6^b^	92.9 ± 0.9^b^	0.1 ± 0.1^a^
Brand1	88.3 ± 0.8^cde^	91.6 ± 0.5^c^	94.8 ± 1.0^cd^	0.3 ± 0.9^b^
Brand2	87.6 ± 1.9^bcd^	91.3 ± 1.0^bc^	94.6 ± 0.3^cd^	0.2 ± 1.2^ab^
Brand3	88.4 ± 0.5^cde^	91.4 ± 0.9^bc^	93.7 ± 0.2^bc^	0.1 ± 0^a^
Brand4	89.8 ± 0.6^e^	92.2 ± 0.4^c^	94.8 ± 0.5^cd^	0.2 ± 0.5^a^
Brand5	80.3 ± 0.7^a^	84.3 ± 0.3^a^	87.8 ± 0.2^a^	0.1 ± 0^a^
Brand6	88.9 ± 0.6^de^	91.8 ± 0.8^c^	94.5 ± 0.5^cd^	0.2 ± 0.3^a^
Brand7	89.0 ± 0.6^de^	91.7 ± 1.0^c^	95.1 ± 1.0^d^	0.1 ± 0.1^a^
Brand8	86.5 ± 0.2^b^	90.2 ± 0.3^b^	93.7 ± 0.1^bc^	0.2 ± 0.1^a^

Mean values with different lower case letters in superscript at the same column are significantly different (p < 0.05).

#### 3.2.5 Thermal stability

**Fig. 8:**
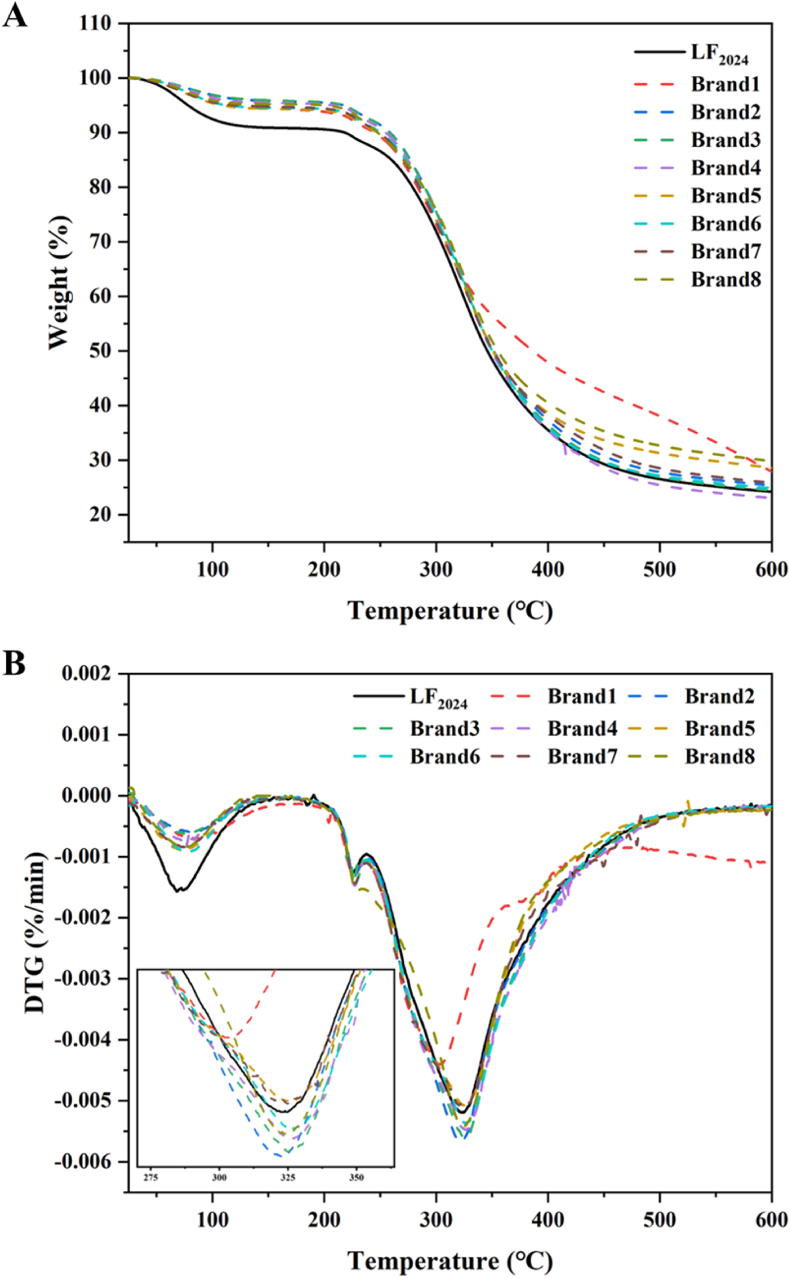
A) TGA and B) DTG curves of LF powders. LF_2024_ is the LF_2016_ sample stored at 4 °C for 9 years. Brands 1–8 are commercial LF powders. (Note: LF_2016_ is not included in this figure for comparison because of the instrument differences).

The TGA and derived thermogravimetric analysis (DTG) curves of the LF powder are presented in Fig. 8. The TGA curve showed significant weight loss in two stages. The first stage occurred at 27°C–150 °C, and the second stage occurred at 150°C–500 °C. The first stage of weight loss was mainly attributable to the evaporation of water from the LF powder (Patil et al., 2020). Rapid weight loss was observed between 150 °C and 500 °C, mainly due to protein degradation (Feroz et al., 2022), that is, degradation of the polypeptide structure. Past studies have indicated that the cleavage of disulphide bonds, along with the liberation of sulfur dioxide and hydrogen sulfide, typically takes place in the temperature range of 230°C–250 °C (Feroz et al., 2022).

LF_2024_ lost more weight in the first stage because it had a higher water content, which reduced its weight by evaporation. With the exception of Brand1, all bLF samples showed similar TGA curves. The thermal weight of LF_2024_ at 600 °C (24.4 %) was lower than that of the commercial LF (23.1 %–29.8 %). This might be related to the covalent complexation of metals during their extraction and production and might also be attributed to more β folds in their molecular structure, which play a role in linking and fixing in molecular interactions (Feroz et al., 2022).

The corresponding DTG curve is shown in Fig. 7B. All LF samples showed significant weight loss, and the peak temperature reflected the thermal stability of the LF samples. In region I (30°C–150 °C) and region II (150°C–600 °C), the mass loss of the sample reflected the loss of free and bound water and the degradation of proteins. In the region I phase, the higher moisture content in LF_2024_ resulted in a higher heat loss compared with other samples. In the region II stage, the commercial LF powder decomposed in the temperature range of 172°C–326 °C and the peak value of the maximum decomposition of LF_2024_ was approximately 323 °C, which are still within the range of commercial bLF powders. The large and sharp peaks in region II are associated with protein degradation. Peak overlaps of the active thermal phase due to protein breakdown have also been reported elsewhere (D. Yu et al., 2020, Yu et al., 2020). These results showed that the long-term storage of bLF at 4 °C and 40 % RH for 9 years did not affect its thermal stability.

### 3.3 Functional activity

#### 3.3.1 Iron content and iron-binding ability

**Fig. 9:**
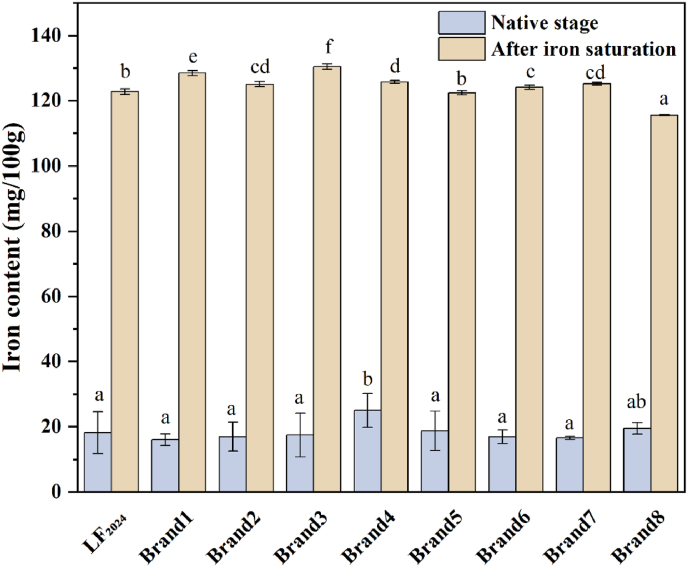
Iron-binding capacity of LF powders. LF_2024_ is the LF_2016_ sample stored at 4 °C for 9 years. Brand 1–Brand 8 are commercial LF powders.

The iron transfer capacity of LF is presented in Fig. 9. The bLF powder (LF_2024_) stored for 9 years had an iron content of 18.2 mg/100 g (Table 3). The iron content of the commercial LF (Brand 1–Brand 8) was in the range of 16.0–25.0 mg/100 g. After reacting with excess iron, the iron content of all bLF samples increased to 115.6–130.4 mg/100 g, equivalent to the iron saturation level of 80.5 %–90.8 %, which can be considered iron-saturated bLF (B. Wang et al., 2017b).

**Table 3:** Iron content and iron-binding ability of LF.

Samples	Iron content (mg/100 g)		
	Initial stage	After iron saturation	Iron binds to LF
LF_2016_	N/A	N/A	N/A
LF_2024_	18.2 ± 6.4^a^	122.5 ± 0.7^b^	104.5 ± 0.8^c^
Brand 1	16.0 ± 1.8^a^	128.5 ± 0.8^e^	112.5 ± 0.8^f^
Brand 2	17.0 ± 4.5^a^	125.1 ± 0.9^cd^	108.1 ± 0.9^de^
Brand 3	17.5 ± 6.7^a^	130.4 ± 0.9^f^	113.0 ± 0.9^f^
Brand 4	25.0 ± 5.2^b^	125.8 ± 0.5^d^	100.8 ± 0.5^b^
Brand 5	18.7 ± 6.0^a^	122.5 ± 0.6^b^	103.7 ± 0.6^c^
Brand 6	16.9 ± 2.1^a^	124.1 ± 0.6^c^	107.2 ± 0.6^d^
Brand 7	16.6 ± 0.5^a^	125.2 ± 0.4^cd^	108.6 ± 0.4^e^
Brand 8	19.5 ± 1.8^ab^	115.6 ± 0.2^a^	96.1 ± 0.2^a^

Mean values with different lower case letters in superscript at the same column are significantly different (p < 0.05).

During long-term storage, the stability of bLF is affected by iron saturation levels and the amount of iron binding in bLF will affect the molecular conformation of protein. It has been reported that LF with iron binding has a more compact structure because the open interdomain cleavage in each leaf of LF is closed in the case of iron ion binding, resulting in a more stable structure. Iron is usually released from LF by three factors: (1) binding to similar specific receptors in the serum, (2) reduction of Fe^3+^ to Fe^2+^ and 3) reduction in pH in the environment (Baker and Baker, 2009). After 9 years of storage, the iron-binding capacity of LF_2024_ (104.5 ± 0.8 mg/100 g) was at the average level of commercial LF powders. Therefore, it is suspected that the iron-binding site of bLF was not damaged during 9 years of storage and its strong iron-binding capacity was reserved.

#### 3.3.2 Effects on antioxidant activity

**Fig. 10:**
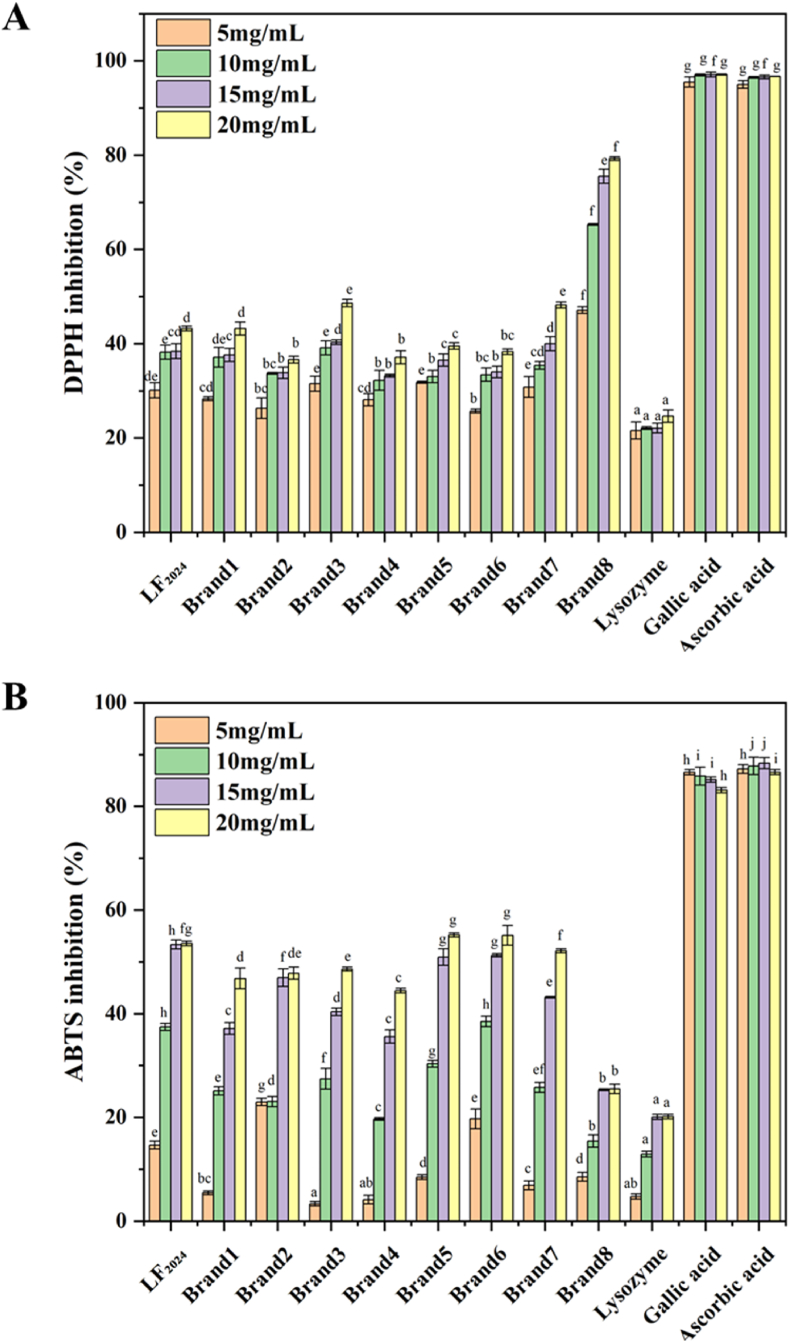
A) Scavenging ability of LF powders on DPPH free radicals and B) scavenging ability of LF powders on ABTS free radicals. LF_2024_ is the LF_2016_ sample stored at 4 °C for 9 years. Brands 1–8 are commercial LF powders. (Note: LF_2016_ is not included in this figure for comparison because of the instrument differences).

The antioxidant capabilities of bLF powders assessed using the DPPH and ABTS assays are presented in Fig. 10. Gallic acid and ascorbic acid, known to have high antioxidant activity, were used as positive controls. Lysozyme, known to have low antioxidant activity, was used as negative control. The antioxidant activities of all samples were in the range of the antioxidant activities of lysozyme and gallic acid/ascorbic acid. With an increase in bLF concentration, the inhibition ability of bLF on DPPH free radicals was gradually enhanced and the inhibition rate was dose dependent. Results showed that the inhibition ability of different bLF samples to DPPH free radicals was significantly different (p < 0.05). In this study, it was found that the DPPH inhibition rates of the bLF powder stored for 9 years (LF_2024_) at concentrations of 5, 10, 15 and 20 mg/mL were 30.1 %, 38.2 %, 38.4 % and 43.2 %, respectively. They were similar to the DPPH inhibition rates of commercial LF samples (36.6 %–48.2 %). The results obtained by ABTS analysis were similar to those obtained by DPPH analysis. The ABTS radical scavenging ability of LF_2024_ at concentrations of 5, 10, 15 and 20 mg/mL were 14.7 %, 37.4 %, 53.3 % and 53.6 %, respectively. It was also similar to the commercially available LF powders. These results indicated that the antioxidant activity of the bLF powders was reserved after 9 years of storage (4 °C and 40 % RH). However, the reduction in antioxidant activity might occur at different storage conditions. If the oxidation of certain amino acid residues in bLF, particularly sulfur-containing amino acids (such as cysteine, tyrosine and phenylalanine), which can directly interact with free radicals (Guo et al., 2024), hereby affecting the protein's structure and function. This oxidation may result in changes in protein folding, increasing the protein's tendency to aggregate and decreasing its biological activity. Previous studies have shown that temperature increase can accelerate the reaction rate in the emulsion, improve the movement of molecules and promote the lipid oxidation process (Zink et al., 2024). In emulsions, unsaturated fatty acids, such as oleic acid and linoleic acid, are more susceptible to oxidation because of their chemical structural properties, forming highly reactive substances such as peroxyradicals. These reactive oxygen species perpetuate reactions with adjacent lipid molecules, triggering a cascade of events in the lipid oxidation process, which ultimately yields a variety of oxidation by-products (Zhang et al., 2021).

#### 3.3.3 Antibacterial activity of LF

**Fig. 11:**
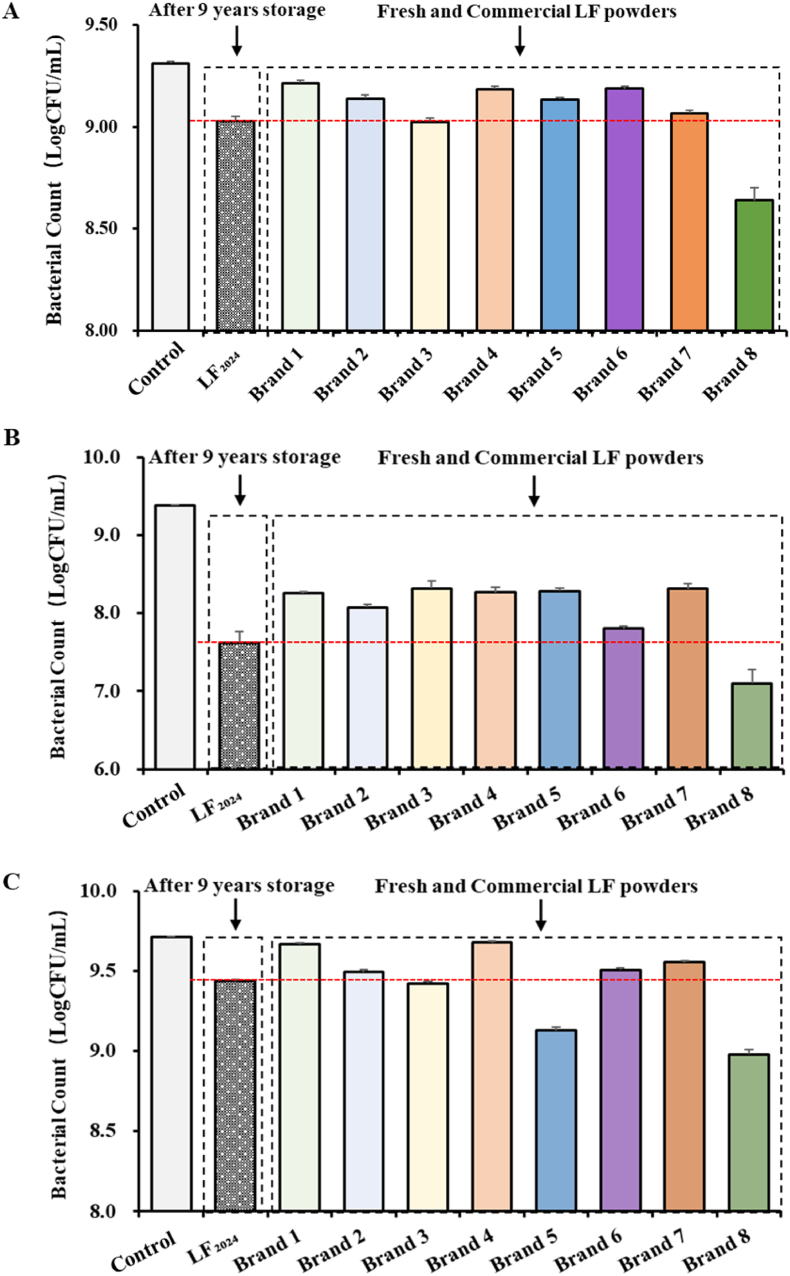
Antibacterial effect of LF against A) *Escherichia coli*, B) *Staphylococcus aureus* and C) *Salmonella enteritidis*. LF addition amount was 18 mg/mL. Culture time was 12 h. Control is the sample that bacteria growth without LF. LF_2024_ is the LF_2016_ sample stored at 4 °C for 9 years. Brands 1–8 are commercial LF powders.

The antibacterial effect of bLF against *E. coli*, *S. aureus* and *S. enteritidis* is shown in Fig. 11. In the presence of LF, the bacterial growth speed was obviously reduced. Therefore, all LF powders showed strong antibacterial effects against *E. coli*, *S. aureus* and *S. enteritidis.* The antibacterial activity of LF was most significant for *S. aureus*, followed by *E. coli* and *S. enteritidis*. Notably, the inhibitory effects of different LF samples were slightly different from each other. This might be due to the different extraction, purification and drying methods applied in these samples.

After 9 years of storage, the freeze-dried bLF powder showed no colony growth (data not shown), indicating that microbial contamination levels were within acceptable limits and that the powder remained stable and sterile throughout the storage period. This finding is significant because it demonstrates that the absence of colony growth indicates that the powder was properly disposed of and stored under conditions that prevent microbial growth. The antibacterial activity of the long-term-stored LF_2024_ against the three types of bacteria was comparable with that of commercial LF products. Interestingly, LF_2024_ showed a higher bacteriostatic effect than most of the commercial bLF samples. This change in antibacterial activity is mainly due to the physicochemical changes that occur over time when the powder is stored in low-temperature, dry and dark environments. The high antibacterial activity of Brand 8 may be due to the fact that it is a recombinant sample. The recombinant LF usually have similar amino acids compositions and protein structures compared to the natural LF. However, the glycoside attached to LF (affect the binding of LF to the cell membrane) showed different interaction behaviours compared to the natural ones (Almond et al., 2013). This could be the reason of the higher antibacterial activity of the Brand8. Besides, Brand 8 is recombinant human LF. The higher antibacterial activity might also be attributed to the different species origin. Although LF_2024_ underwent significant physicochemical changes during long-term storage, it did not lose its antimicrobial ability compared with commercial LF at the end of the study. This may be because the increase in water molecules of LF_2024_ causes the hydration layer around the protein molecules to thicken. This hydration may alter the hydrogen bonding in the protein molecule, which, in turn, affects the protein's folded form (Makhatadze and Privalov, 1993). In some cases, hydration may prompt the protein to unfold, exposing hydrophobic amino acid residues that would otherwise on the inside of the protein's surface. These residues can interact with the lipid portion of the bacterial cell wall, enhancing protein binding to the bacteria (Pandit et al., 2021). Overall, this trial has shown that the long-term stored bLF exhibits antibacterial properties against various pathogens such as *E. coli*, *S. aureus* and *S. enteritidis*, consistent with the findings of previous studies (Sawale et al., 2024).

#### 3.3.4 Haemolysis test

**Fig. 12:**
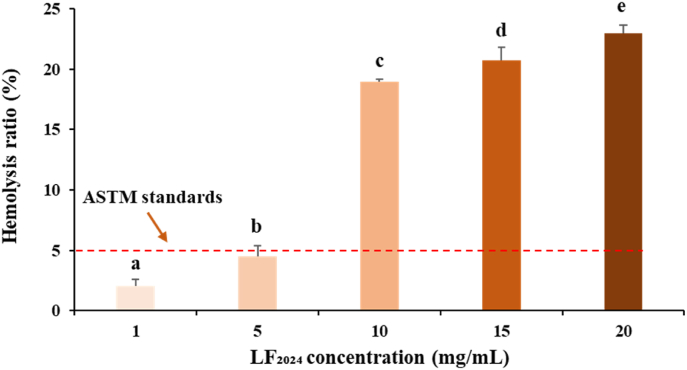
Haemolysis rates of red blood cells incubated with the 9-year-stored bLF.

The biocompatibility assessed by the haemolysis test is presented in Fig. 12. The defibrillated sheep blood was incubated with the bLF powder stored for 9 years (LF_2024_). It was found that the haemolysis rate was positively correlated with the bLF concentration. The haemolysis rates corresponding to 1 and 5 mg/mL bLF incubation were 2.04 % and 4.50 %, respectively, meeting the ASTM standards of good biocompatibility (<5 %, ASTM F756-2008) (Huang et al., 2016). When the bLF concentration was increased to 10, 15 and 20 mg/mL, the corresponding haemolysis rates reached 18.97 %, 20.74 % and 22.99 %, respectively, indicating that significant haemolysis occurred. Therefore, bLF stored for 9 years did not exhibit acute haemolytic reaction in contact with blood at a concentration of <5 mg/mL. This is a regular concentration range of bLF addition in many food and biological products (Hu et al., 2024; Jiang et al., 2024). Therefore, the haemolysis test implies that the freeze-dried bLF powder still possesses good biocompatibility after 9 years of storage.

## 4 Conclusions

Dairy industries have claimed up to 5 years of shelf life of bLF powders. This study extended the storage period to 9 years and reported that bLF powders could be more self-stable than documented in literature. After 9 years of storage at 4 °C and 40 % RH environment, there were minimal changes in the physicochemical characteristics, structural features and functional properties of bLF powders, although the moisture content of bLF increased from 2.7 % to 8.8 % with powder cracks. The 9-year-stored bLF powders demonstrated comparable DPPH and ABTS free radical scavenging capabilities to those of commercial LF powders. The iron-binding capacity (104.5 mg/100 g) was also similar to that of commercial LF powders (96.1–113 mg/100 g). Furthermore, bLF did not exhibit bacterial invasion during the long-term storage. After 9 years of storage, the antibacterial activity against *E. coli*, *S. aureus* and *S. enteritidis* was even higher than that of commercial LF powders. Therefore, bLF remains stable and functionally active as an antioxidant, iron transporter and antibacterial agent after 9 years of storage (4 °C and 40 % RH). Overall, this study provides fundamental support for the long-term storage stability of the bLF powder. It also emphasises the potential application of bLF powders in the advancement of aerospace food, e.g. extending the shelf life of aerospace foods, especially during long-term space missions.

## CRediT authorship contribution statement

**Yin Hu:** Methodology, Investigation, Visualization, Writing – original draft, Formal analysis. **Shubo Luo:** Methodology, Investigation, Visualization, writing. **Yuhong Jiang:** Methodology, Investigation, Visualization. **Jie Lin:** Methodology, Investigation, Formal analysis. **Baoguo Xu:** Methodology, Investigation, Validation. **Zhi-Hong Zhang:** Methodology, Investigation, Writing – review & editing. **Benu Adhikari:** Methodology, Investigation, Writing – review & editing, Formal analysis. **Tiantian Xu:** Supervision, Conceptualization, Validation, Writing – review & editing. **Bo Wang:** Supervision, Conceptualization, Project administration, Validation, writing.

## Declaration of competing interest

Competing interests: Shubo Luo is a current employee of China Heilongjiang Feihe Dairy Co., Ltd. Yuhong Jiang and Jie Lin are current employees of Nanjing Bestzyme Bio-Engineering Co., Ltd.

## Data Availability

Data will be made available on request.
