# Supplementary material for: Stability and functionality of bovine lactoferrin powder after 9 years of storage
Source: Curr Res Food Sci. 2025 Mar 18;10:101036. doi: 10.1016/j.crfs.2025.101036 (PMC11981779; doi:10.1016/j.crfs.2025.101036)
Supplement: Multimedia component 1 [file mmc1.docx]

**Table S1.** Physicochemical properties of different LF powders. Kjeldahl method was used to determine the protein content of LF.

| Samples | Moisture content  (%) | Protein content (%) | Water solubility (%) | Colour | | |
| --- | --- | --- | --- | --- | --- | --- |
|  |  |  |  | L^*^ | a^*^ | b^*^ |
| **LF_2016_** | 2.7 ± 0.5^a^ | N/A | 93.5 ± 0.1^abc^ | 72.9 ± 1.6^ab^ | 8.8 ± 0.6^c^ | 15.7 ± 0.8^cde^ |
| **LF_2024_** | 8.8 ± 0.8^b^ | 95.7±0.7^c^ | 93.0 ± 1.7^abc^ | 72.9 ± 0.1^bcd^ | 10.9 ± 0.2^de^ | 16.8 ± 0.2^de^ |
| Brand1 | 2.8 ± 1.7^a^ | 96.7±0.2^c^ | 93.5 ± 0.9^abc^ | 68.1 ± 0.6^a^ | 11.4 ± 0.7^e^ | 17.2 ± 0.5^e^ |
| Brand2 | 3.2 ± 1.9^a^ | 97.3±0.7^cd^ | 91.9 ± 2.1^a^ | 72.9 ± 0.8^bc^ | 9.9 ± 0.2^cde^ | 16.5 ± 0.3^de^ |
| Brand3 | 2.6 ± 1.9^a^ | 98.0±0.4^de^ | 92.7 ± 0.7^ab^ | 77.0 ± 2.9^def^ | 7.2 ± 0.1^b^ | 13.0 ± 0.2^ab^ |
| Brand4 | 3.5 ± 1.5^a^ | 96.9±0.8^c^ | 95.3 ± 0.3^c^ | 73.2 ± 0.5^bcde^ | 9.5 ± 0.7^cd^ | 15.9 ± 0.4^cde^ |
| Brand5 | 4.1 ± 1.6^a^ | 89.4±0.5^a^ | 94.0 ± 1.4^abc^ | 79.3 ± 1.2^f^ | 6.6 ± 1.2^ab^ | 14.3 ± 0.6^bc^ |
| Brand6 | 5.3 ± 0.3^a^ | 95.4±0.6^b^ | 93.3 ± 1.3^abc^ | 77.2 ± 1.7^ef^ | 7.6 ± 1.6^bc^ | 14.6 ± 1.1^cd^ |
| Brand7 | 3.0 ± 1.4^a^ | 98.1±0.3^de^ | 92.9 ± 1.9^ab^ | 84.7 ± 0.8^g^ | 6.6 ± 0.4^a^ | 14.9 ± 0.4^cd^ |
| Brand8 | 4.0 ± 1.3^a^ | 98.7±0.6^e^ | 94.5 ± 0.5^bc^ | 76.0 ± 1.8^cdef^ | 6.9 ± 0.3^ab^ | 12.4 ± 0.2^a^ |

Mean values with different lower case letters in superscript at the same column are significantly different (p < 0.05).
